# Heterologous Prime-Boost Vaccination with Inactivated Influenza Viruses Induces More Effective Cross-Protection than Homologous Repeat Vaccination

**DOI:** 10.3390/vaccines11071209

**Published:** 2023-07-06

**Authors:** Noopur Bhatnagar, Ki-Hye Kim, Jeeva Subbiah, Sakinah Muhammad-Worsham, Bo Ryoung Park, Rong Liu, Phillip Grovenstein, Bao-Zhong Wang, Sang-Moo Kang

**Affiliations:** 1 Center for Inflammation, Immunity & Infection, Institute for Biomedical Sciences, Georgia State University, Atlanta, GA 30302, USA; nbhatnagar1@gsu.edu (N.B.); kkim39@gsu.edu (K.-H.K.); tof6@cdc.gov (J.S.); smuhammadworsham1@student.gsu.edu (S.M.-W.); bpark9@gsu.edu (B.R.P.); rliu12@student.gsu.edu (R.L.); pgrovenstein1@student.gsu.edu (P.G.); bwang23@gsu.edu (B.-Z.W.); 2 Influenza Division, National Center for Immunization and Respiratory Diseases, Centers for Disease Control and Prevention, Atlanta, GA 30329, USA

**Keywords:** influenza virus, inactivated virus vaccine, heterologous prime-boost vaccination, cross-protection

## Abstract

With concerns about the efficacy of repeat annual influenza vaccination, it is important to better understand the impact of priming vaccine immunity and develop an effective vaccination strategy. Here, we determined the impact of heterologous prime-boost vaccination on inducing broader protective immunity compared to repeat vaccination with the same antigen. The primed mice that were intramuscularly boosted with a heterologous inactivated influenza A virus (H1N1, H3N2, H5N1, H7N9, H9N2) vaccine showed increased strain-specific hemagglutination inhibition titers against prime and boost vaccine strains. Heterologous prime-boost vaccination of mice with inactivated viruses was more effective in inducing high levels of IgG antibodies specific for groups 1 and 2 hemagglutinin stalk domains, as well as cross-protection, compared to homologous vaccination. Both humoral and T cell immunity were found to play a critical role in conferring cross-protection by heterologous prime-boost vaccination. These results support a strategy to enhance cross-protective efficacy by heterologous prime-boost influenza vaccination.

## 1. Introduction

Influenza viruses cause 290,000–640,000 deaths per year globally [1,2]. Influenza A virus has high antigenic diversity, originating from 18 hemagglutinin (HA) subtypes (H1–H18) and 11 neuraminidase (NA) subtypes (N1–N11). The HA subtypes of influenza A virus are divided into two main phylogenetic groups: group 1 (G1), which includes H1, H2, H5, H6, H8, H9, H11, H12, H13, H16, H17, and H18; and group 2 (G2), which includes H3, H4, H7, H10, H14, and H15 (Supplementary Figure S1A) [3]. Influenza B virus has evolved into antigenically distinct Victoria and Yamagata lineage strains. Current influenza vaccines are either trivalent (containing H1N1, H3N2, and one influenza B virus strain) or quadrivalent (containing influenza A H1N1 and H3N2 as well as both lineages of influenza B virus strains). The vaccine components can be the same strains used in previous years, or some vaccine strains are updated annually to better reflect the circulating influenza strains. Annual repeat vaccinations are recommended as a preventive measure, but they result in variable effectiveness against influenza [4,5,6,7]. Due to the continuous emergence of drift mutations and pandemics, the overall effectiveness after seasonal vaccination has been in a low range (between 10% and 60%) during the last few decades [8,9]. During the 2014–2015 season, the efficacy of the H3N2 vaccine component was estimated to be as low as 6%, partially due to drift mutations in the circulating H3N2 strains [10].

There are controversial studies reporting that annual repeat influenza vaccination does not improve protective immune responses [11,12], lowers vaccine efficacy against mismatched strains [13], and fails to increase antibody affinity maturation in humans [12,14,15]. Pre-existing immunity shapes immune responses toward earlier influenza antigens or conserved domains [16,17]. It has been a high priority to better understand how prior immune responses modulate cross-protective immune responses to subsequent vaccination and enhance vaccine efficacy toward broader protection.

In this study, we tested a hypothesis that a strategy of heterologous prime-boost vaccination with antigenically diverse inactivated influenza viruses would induce broader protective immune responses than repeat vaccination with the same antigen in a mouse model. Heterologous or heterosubtypic boost immunization with antigenically different virus vaccines from the strain used for priming was found to enhance HAI activities against the prime vaccine strain and heterologous boost viruses as well as cross-protection. Possible correlates of group-specific HA stalk IgG antibody responses and the roles of humoral and cellular immunity were also investigated.

## 2. Materials and Methods

### 2.1. Animals, Reagents, and Viruses

Adult BALB/c mice (6- to 8-week-old, female) were purchased from Jackson Laboratory (Bar Harbor, ME, USA) and maintained in the animal facility at Georgia State University (GSU). All mouse studies were approved by GSU Institutional Animal Care and Use Committee (IACUC, A21004) and carried out in compliance with the Guide for the Care and Use of Laboratory Animals of the NIH.

Groups 1 (G1) and 2 (G2) inactivated influenza viruses were used for immunizations and as coating antigens for enzyme-linked immunosorbent assay (ELISA). The inactivated viruses were prepared as previously described [18]. Briefly, G1 and G2 influenza viruses were inactivated with 1% formalin and concentrated by ultracentrifugation (SW32 Ti rotor, 123,760× *g*, 1 h). The inactivated virus pellet was resuspended in phosphate-buffered saline (PBS) and the total protein concentration of the inactivated viruses was determined using the DC protein assay kit (Bio-Rad Laboratories, Hercules, CA, USA). The G1 inactivated influenza A viruses were as follows: A/Puerto Rico/8/1934 H1N1 (iPR8/H1N1), A/California/04/2009 H1N1 (iCal/H1N1), A/Indonesia/5/2005 H5N1 (iIndo/H5N1), and A/Hong Kong/1073/1999 H9N2 (iHK/H9N2). The G2 inactivated viruses were as follows: A/Hong Kong/1/1968 H3N2 (iHK/H3N2) and reassortant A/Shanghai/11/2013 H7N9 with A/Puerto Rico/8/1934 (A/PR8) H1N1 backbone (iSH/H7N9) [19,20]. The Challenge viruses included G2 (A/Philippine/2/1982 H3N2 (Phil/H3N2)) and G1 (A/Hong Kong/1073/1999 H9N2 (HK/H9N2)) viruses and reverse genetics (rg) reassortant H5N1 (rgH5N1) virus containing HA and NA derived from A/Vietnam/1203/2004 and six internal genes from A/PR8 H1N1, as previously described [21]. Phil/H3N2, HK/H9N2, and rgH5N1 viruses were propagated using embryonated chicken eggs.

### 2.2. Immunization and Virus Challenge of Mice

Groups of 6-week-old BALB/c mice (*n* = 5) were intramuscularly immunized with 5 µg (total proteins) of a specific strain of inactivated influenza virus and then boosted with 5 µg of homologous, heterologous, or heterosubtypic inactivated virus (H1N1, H3N2, H5N1, H7N9, H9N2)/mouse at 3-week intervals. The prime-boost vaccine strains consisted of iPR8/H1N1-iPR8/H1N1, iPR8/H1N1-iCal/H1N1, iCal/H1N1-iPR8/H1N1, iPR8/H1N1-iHK/H3N2, iHK/H3N2-iPR8/H1N1, iPR8/H1N1-iIndo/H5N1, iIndo/H5N1-iPR8/H1N1, iPR8/H1N1-iSH/H7N9, iSH/H7N9-iPR8/H1N1, iPR8/H1N1-iHK/H9N2, and iHK/H9N2-iPR8/H1N1. Blood samples were collected 2 weeks after immunizations, and sera were separated to analyze virus- and HA stalk-specific IgG antibody levels as well as HAI titers. Six to eight weeks after boost vaccination, the mice were challenged with a lethal dose of either Phil/H3N2 virus (6.7 × LD_50_), or HK/H9N2 virus (6.7 × LD_50_), or rgH5N1 virus (5 × LD_50_). After challenge, body weight changes and survival rates were monitored for 14 days, and lung viral titers and detailed immunological profiles were determined in mediastinal lymph nodes (MLN) and spleen tissues collected on day 6 post-infection.

### 2.3. Antibody Enzyme-Linked Immunosorbent Assay (ELISA)

To measure antigen-specific antibody levels, inactivated viruses (200 ng/well) were coated onto ELISA plates and then incubated with diluted immune sera, as previously detailed [22]. The relative IgG antibody levels in serially diluted sera were presented as optical density absorbance values at 450 nm (BioTek ELISA plate reader) or concentrations as calculated using standard IgG (Southern Biotechnology, Birmingham, AL, USA). Additionally, consensus G1 and G2 HA2 stalk proteins, prepared as previously described [23], were used as ELISA coating antigens (50 ng/well) to determine HA2 stalk-specific IgG antibody levels.

### 2.4. Hemagglutination Inhibition (HAI) Assay

To determine HAI titers in immune sera, the serum samples were treated with receptor-destroying enzyme (RDE, Sigma-Aldrich, St. Louis, MO, USA), followed by inactivation (56 °C, 30 min) and mixing with an equal volume of 4 HA units of target viruses. HAI titers were determined as the highest dilution factor inhibiting the formation of buttons with 0.5% chicken red blood cells (RBC, Lampire Biological Laboratories, Pipersville, VA, USA), as previously described [24].

### 2.5. Lung Viral Titration

Lung extracts, prepared in 1.5 mL of Roswell Park Memorial Institute (RPMI) 1640 by mechanically grinding lung tissues harvested on day 6 after challenge, were used to determine viral titers in embryonated chicken eggs (Hy-Line North America, LLC., Mansfield, GA, USA), as previously described [25]. Virus titers as 50% egg infectious dose (EID_50_)/mL were evaluated according to the Reed and Muench method [26].

### 2.6. In Vitro IgG Antibody Detection and Cytokine ELISA

Lung extracts were used to determine in vitro G1 and G2 HA stalk-specific antibody levels. Levels of inflammatory cytokines, including tumor necrosis factor (TNF)-α, interleukin (IL)-6, interferon (IFN)-γ, and IL-1β, in the lung extracts were measured by cytokine ELISA, as previously described [27]. Cytokine levels were detected using the Ready-SET-Go kit with TNF-α-, IL-6-, IFN-γ-, or IL-1β-specific antibodies (eBioscience, San Diego, CA, USA).

Secreted IgG antibodies specific for G1 and G2 HA stalk proteins were determined in MLNs (5 × 10^5^ cells/well) from mice. The cells from MLNs were isolated on day 6 post-infection and cultured for 5 days in plates pre-coated with G1 and G2 HA stalk proteins. The combined levels of IgG antibodies secreted into the culture supernatants and those captured on the plate were analyzed by ELISA.

### 2.7. In Vivo Protection Efficacy Test of Immune Sera

Immune sera collected two weeks after boost immunization were diluted 4-fold, heat-inactivated at 56 °C for 30 min, followed by mixing with the same volume of A/Phil/H3N2 virus (3 × LD_50_) or rgH5N1 virus (3 × LD_50_) and incubation at room temperature for 30 min, as previously described [28]. The mixture of A/Phil/H3N2 virus or rgH5N1 virus and sera was intranasally administered to naïve BALB/c mice (*n* = 4 per group), and body weight changes and survival rates were monitored daily for 14 days.

### 2.8. In Vivo Depletion of T Cells

For in vivo systemic T cell depletion before and post-challenge, prime-boost immunized BALB/c mice (*n* = 4) received treatment with anti-CD4 (CD4 clone GK1.5) or anti-CD8 (CD8 clone 53.6.7) monoclonal antibodies (mAbs), as previously described [19]. Antibodies (BioXCell, West Lebanon, NH, USA) were injected into the mice via intraperitoneal (IP; 2 days before and after challenge) and intranasal (IN; 2 days after challenge) sequential delivery at 4-day intervals (200 μg anti-CD4 and 150 μg anti-CD8/mouse for IP injection, 10 μg anti-CD4/8/mouse for IN inoculation). All groups were challenged with a lethal dose of A/Phil/H3N2 influenza virus (3 × LD_50_), and body weight changes and survival rates were monitored daily for 14 days after challenge.

### 2.9. Intracellular Cytokine Staining and Flow Cytometry Analysis

Lung cells were harvested from the layer between 44% and 67% Percoll and stimulated with 4 μg/mL inactivated A/Cal H1N1 or A/SH H7N9 virus in the presence of Brefeldin A (20 μg/mL) for 5 h at 37 °C, as previously described [18]. In vitro cultured lung cells were stained with anti-CD3-PacificBlue (Clone 17A2, Biolegend, San Diego, CA, USA) and anti-CD4-PE/Cy5 (Clone RM405, BD Biosciences, San Jose, CA, USA) antibodies, and then fixed and permeabilized using the BD Cytofix/Cytoperm^TM^ Plus Kit (BD Biosciences). After staining the cells with anti-IFN-γ-APC/Cy7 antibodies (Clone XMG1.2, BD), live lymphocytes were first gated by forward versus side scatter strategic gating, followed by gating of CD3^+^ T cells and then gating of CD4 T cells secreting cytokines. The number of effector T cells in lung extract were expressed by reflecting their frequency gated on the total cells from each mouse. Cells positive for intracellular cytokines were revealed through acquisition on a Becton-Dickinson LSR-II/Fortessa flow cytometer (BD, San Diego, CA, USA) and analyzed by Flowjo software (Tree Star Inc., Ashland, OR, USA).

### 2.10. Statistical Analyses

All results are presented as mean ± standard error of the mean (SEM). The statistical significance for all the experiments was calculated by one-way or two-way analysis of variance (ANOVA). A *p*-value < 0.05 was considered significant. Data analysis was performed using Prism software (GraphPad Software Inc., San Diego, CA, USA).

## 3. Results

### 3.1. Heterologous and Heterosubtypic Prime-Boost Vaccinations Induce Cross-Reactive Virus-Specific IgG and Group-Specific HA Stalk Antibodies

**Figure 1:**
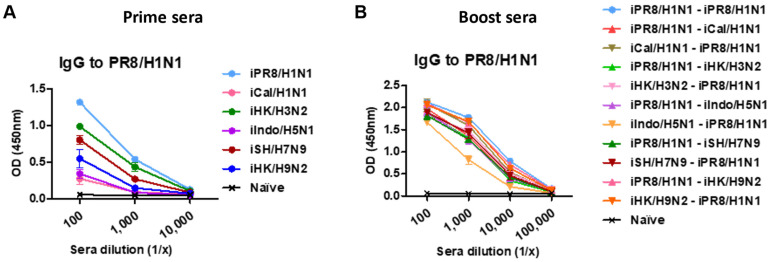
A/PR8/H1N1 virus-specific IgG antibody responses in prime or homologous and heterologous prime-boost immune sera. BALB/c mice (*n* = 5 per group) were intramuscularly primed with 5 µg of a specific strain of inactivated influenza virus and then boosted with 5 µg (per mouse) of homologous, heterologous, or heterosubtypic inactivated virus (H1N1, H3N2, H5N1, H7N9, H9N2) at 3-week intervals. (**A**) Relative comparison of PR8/H1N1 virus-specific ELISA IgG antibodies in antisera after prime immunization with different inactivated viruses as indicated in each group. (**B**) PR8/H1N1 virus-specific ELISA IgG antibody levels in boost sera after homologous and heterologous prime-boost vaccination as indicated in the group labels. Relative comparison of A/PR8 virus-specific IgG antibody responses is presented as optical density (OD) values of ELISA readings.

We first analyzed the phylogenetic distance and amino acid (aa) homology of the viruses used in this study, highlighting the HA sequence diversity within the same group and between groups 1 and 2 HA viruses (Supplementary Figure S1A–C). To study the impact of pre-existing immunity, groups of BALB/c mice were intramuscularly (I.M.) primed with a specific strain of inactivated influenza virus and then boosted with homologous, heterologous, or heterosubtypic inactivated virus (H1N1, H3N2, H5N1, H7N9, H9N2). Prime immunization with a strain of inactivated virus induced IgG responses specific for that prime strain. A similar pattern of antigen-specific IgG antibody responses was observed with other prime immunizations (Table 1). For example, iPR8/H1N1-primed mice induced the highest levels of iPR8/H1N1 virus-specific IgG antibodies (Figure 1A), and iCal/H1N1-primed mice induced the highest levels of iCal/H1N1 virus-specific IgG antibodies. Also, the prime groups exhibited vaccine-specific group 1 (G1) or group 2 (G2) stalk-specific IgG antibodies. The iPR8/H1N1- and iCal/H1N1-primed groups exhibited moderate levels of G1 stalk-specific IgG antibodies whereas the iIndo/H5N1- and iHK/H9N2-primed groups exhibited low levels of G1 stalk-specific IgG antibodies. Also, the iHK/H3N2- and iSH/H7N9-primed groups exhibited low levels of G2 stalk-specific IgG antibodies. Boost immunizations enhanced the levels of IgG antibodies that were highly cross-reactive to different heterologous and heterosubtypic viruses (Figure 1B and Table 2). Also, G1 and G2 stalk-specific IgG levels were highly boosted after prime-boost vaccinations. Specifically, the iPR8/H1N1-iSH/H7N9 and iSH/H7N9-iPR8/H1N1 heterologous prime-boost vaccination groups exhibited the highest levels of IgG antibodies for homologous, heterologous, and heterosubtypic viruses, as well as G1 and G2 HA stalk domains. These results suggested that heterosubtypic prime-boost vaccination induced cross-reactive IgG antibodies to different virus subtypes and both G1 and G2 HA2 stalk domains.

### 3.2. Heterologous Boost Enhances the Induction of HAI Titers against Prior Prime and Current Boost Virus Strains

**Figure 2:**
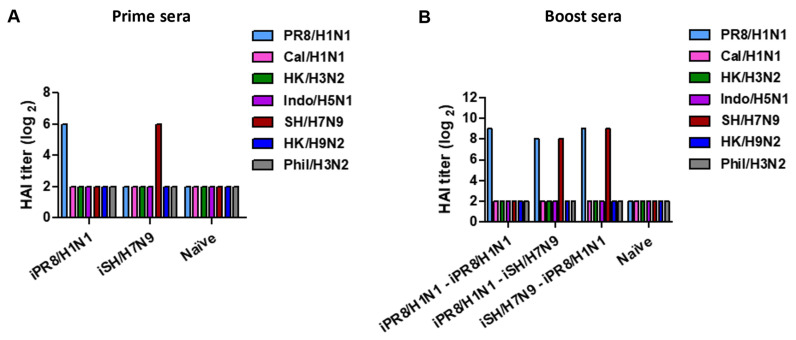
Representative data of HAI titers in immune sera collected after prime vaccination or homologous and heterologous prime-boost vaccinations. The immunization scheme (*n* = 5 per group) was the same as in Figure 1. Blood samples were collected 2 weeks after prime and boost immunizations, and sera were collected to analyze HAI titers. (**A**) HAI titers against different viruses are indicated as color bars in prime sera from the representative inactivated virus prime vaccination (iPR8/H1N1, iSH/H7N9). (**B**) HAI titers against different viruses as indicated in boost sera from representative homologous and heterologous vaccine groups.

We measured HAI titers as a protective immune correlate in antisera collected 2 weeks after prime and boost immunizations with inactivated viruses. Prime immunization with inactivated viruses induced strain-specific HAI titers but not cross-reactive HAI activities (Figure 2A and Table 3). A wide range of HAI titers was observed in the prime iIndo/H5N1 group displaying a low HAI titer of 1:32 and the iHK/H3N2 and iHK/H9N2 groups displaying high HAI titers of 1:256 (Table 3), suggesting differential induction of HAI antibodies by different inactivated viruses. After boost vaccination, the iPR8/H1N1-iPR8/H1N1 homologous group exhibited an 8-fold increase in HAI titers against PR8/H1N1 virus, whereas heterologous or heterosubtypic boost induced 2- to 8-fold increases in HAI titers against both prime and boost strains (Figure 2B and Table 4). It is notable that the hetero prime-boost groups exhibited HAI titers against both viruses used as prime and boost vaccines at higher levels (Table 4) in most cases than those after the prime dose (Table 3). Also, no cross-reactive HAI antibodies were induced after boost immunization. These results suggested that heterosubtypic prime-boost vaccination can effectively induce HAI activities against the heterologous strains used for prime and boost and that primed immunity can promote the induction of HAI antibodies against both prime and boost viruses.

### 3.3. Heterologous and Heterosubtypic Prime-Boost Vaccinations Induce Cross-Protection

**Figure 3:**
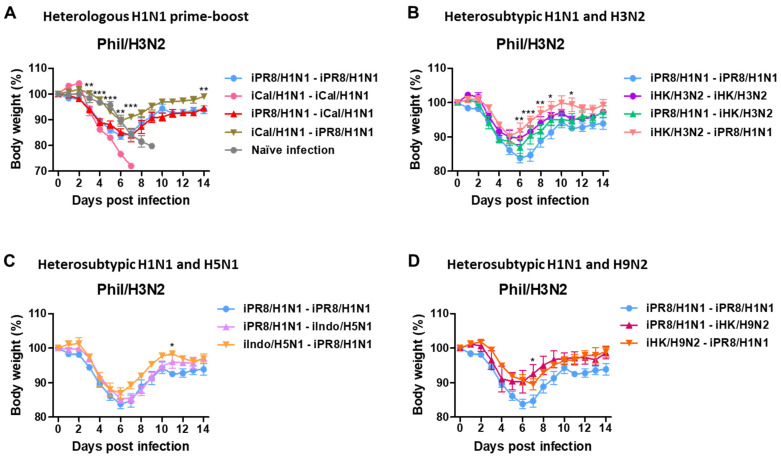
Body weight changes in the homologous and heterologous prime-boost groups after challenge with Phil/H3N2 virus. Immunization and challenge scheme (*n* = 5 mice per group) with the same vaccine groups as in Figure 1. Eight weeks post-boost immunization, the mice were challenged with a lethal dose of Phil/H3N2 virus (6.7 × LD_50_, equivalent to 9.4 × 10^2^ EID_50_). (**A**–**D**) Body weight changes were monitored for 14 days. Statistical significance was calculated using two-way ANOVA and Bonferroni’s post-multiple comparison test. Error bars indicate the mean ± SEM. *; *p* < 0.05, **; *p* < 0.01, ***; *p* < 0.001, compared to the iPR8/H1N1-iPR8/H1N1 group.

To investigate the cross-protective efficacy, mice were challenged with Phil/H3N2 virus at 8 weeks after boost immunization. After infection with Phil/H3N2 virus, naïve mice showed severe weight loss of over 21%, reaching the endpoint with no survival (Figure 3A). The H1N1 homologous prime-boost group iCal/H1N1-iCal/H1N1 did not survive, and the iPR8/H1N1-iPR8/H1N1 group displayed a severe weight loss of 16.2%. The iPR8/H1N1-iCal/H1N1 group exhibited a similarly severe weight loss (~16%), but the reverse order iCal/H1N1-iPR8/H1N1 group displayed a moderate weight loss of 10%, in comparison to the iCal/H1N1-iCal/H1N1 group that did not survive the challenge. The heterosubtypic H1N1 and H3N2 prime-boost groups (iPR8/H1N1-iHK/H3N2 and iHK/H3N2-iPR8/H1N1) displayed weight losses of ~13% and 9%, respectively, and the homologous iHK/H3N2 prime-boost group showed a weight loss of ~10% (Figure 3B). The low weight loss in the iHK/H3N2- iHK/H3N2 group might have been because both the inactivated viruses used for prime-boost immunization and the challenge virus belonged to the same H3N2 subtype. The heterosubtypic H1N1 and H5N1 prime-boost groups (iPR8/H1N1-iIndo/H5N1 and iIndo/H5N1-iPR8/H1N1) showed weight losses of ~14% and 13%, respectively (Figure 3C) and the heterosubtypic H1N1 and H9N2 prime-boost groups (iPR8/H1N1-iHK/H9N2 and iHK/H9N2-iPR8/H1N1) showed weight losses of ~9% and 10%, respectively (Figure 3D). These results suggested that phylogenetically distant heterologous prime-boost vaccinations could be more effective in conferring cross-protection against Phil/H3N2 virus than homologous vaccination.

### 3.4. H1N1 and H7N9 Prime-Boost Vaccination Provides Enhanced Heterosubtypic Protection against H3N2 Virus, Supporting a Correlation with Stalk Antibodies

**Figure 4:**
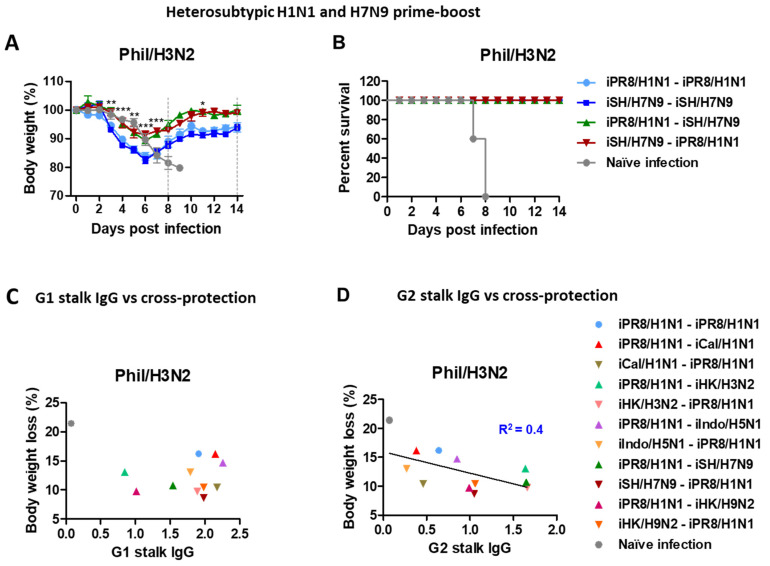
Body weight changes in the homologous and heterosubtypic iSH/H7N9-iPR8/H1N1 groups after Phil/H3N2 challenge and data plots for a possible correlation between HA stalk IgG levels and body weight loss. Immunization (*n* = 5 mice per group) with the same vaccine groups as in Figure 1 in addition to iCal/H1N1-iCal/H1N1, iHK/H3N2-iHK/H3N2 and iSH/H7N9-iSH/H7N9 prime-boost vaccine groups. Eight weeks post-boost immunization, the mice were challenged with the same dose of Phil/H3N2 virus (6.7 × LD_50_, equivalent to 9.4 × 10^2^ EID_50_) as in Figure 3. (**A**,**B**) Body weight changes were monitored for 14 days. (**C**) Levels of G1 stalk-specific and (**D**) G2 stalk-specific IgG antibodies from boost sera were plotted against body weight loss percentages in the different vaccine groups after Phil/H3N2 virus challenge. A line represents the correlation with a coefficient of determination (R^2^) value of 0.4. Statistical significance was calculated using two-way ANOVA and Bonferroni’s post-multiple comparison test. Error bars indicate the mean ± SEM. *; *p* < 0.05, **; *p* < 0.01, ***; *p* < 0.001, comparison between iSH/H7N9-iSH/H7N9 and iSH/H7N9-iPR8/H1N1 groups.

The heterosubtypic iPR8/H1N1-iSH/H7N9 and iSH/H7N9-iPR8/H1N1 groups displayed only ~10% and 8% weight losses, respectively, with 100% survival rates (Figure 4A,B). In contrast, the homologous iSH/H7N9-iSH/H7N9 and iPR8/H1N1-iPR8/H1N1 groups displayed severe weight losses (~17%). These results provided additional evidence that the heterosubtypic H1N1 and H7N9 prime-boost vaccinations were more effective in inducing cross-protection against A/Phil/H3N2 virus than homologous repeat vaccination.

We also evaluated the possibility of a correlation between levels of HA stalk-specific antibodies from boost sera and protection against Phil/H3N2 virus challenge. The data supported a moderate correlation between the G2 (but not G1) HA stalk-specific IgG levels and the efficacy of protection against Phil/H3N2 virus with a coefficient of determination (R^2^) value of 0.4 (Figure 4C,D).

### 3.5. Immune Sera and T Cells Contribute to Protection against Phil/H3N2 Virus after H7N9-H1N1 Prime-Boost Vaccination

**Figure 5:**
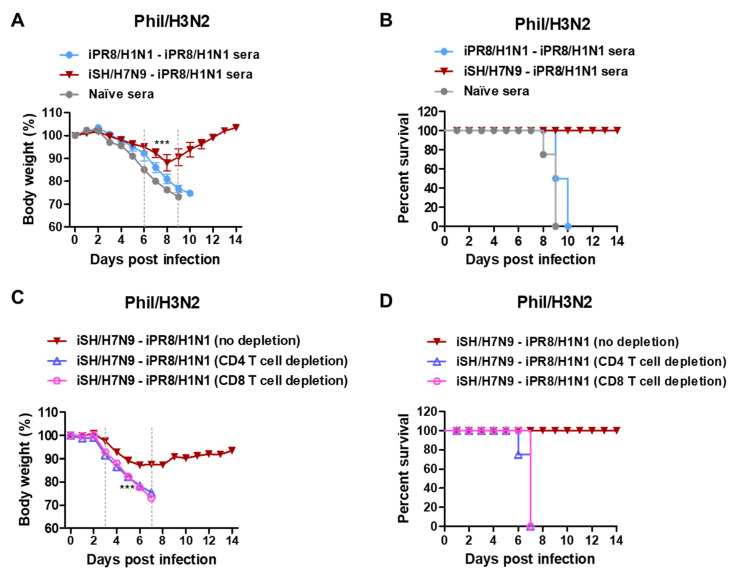
Immune sera and T cells from iSH/H7N9-iPR8/H1N1 prime-boost vaccination contribute to protection against A/Phil/H3N2 virus. (**A**) Body weight changes and (**B**) survival rates were monitored for 14 days in naïve mice (*n* = 4 per group) after inoculation with a mixture of Phil/H3N2 virus (3 × LD_50_, equivalent to 2.3 × 10^2^ EID_50_) and boost immune sera. (**C**) Body weight changes and (**D**) survival rates in the iSH/H7N9-iPR8/H1N1 prime-boost immunized BALB/c mice (*n* = 4) with CD4 or CD8 T cells depleted and Phil/H3N2 virus challenge. Statistical significance was calculated using two-way ANOVA and Bonferroni’s post-multiple comparison test. Error bars indicate the mean ± SEM. ***; *p* < 0.001 compared to the naïve sera group in (**A**) and no depletion group in (**C**).

To determine the roles of humoral immune antisera in conferring heterosubtypic cross-protection, naïve mice were intranasally inoculated with a mixture of A/Phil/H3N2 virus at a lethal dose and antisera that were collected from vaccinated or naïve mice. Naïve sera did not provide protection against Phil/H3N2 virus, as evidenced by severe weight loss (>25%) and 0% survival rate in naïve mice (Figure 5A,B). In contrast, immune sera from the heterosubtypic iSH/H7N9-iPR8/H1N1 group conferred protection in naïve mice with moderate weight loss (~11%) and 100% survival rate. Meanwhile, antisera from the homologous iPR8/H1N1-iPR8/H1N1 group did not provide protection to naïve mice, as displayed by severe weight loss (>25%). These data suggested that humoral responses from heterosubtypic iSH/H7N9-iPR8/H1N1 vaccination significantly contributed to protection against Phil/H3N2 virus.

To investigate whether T cell immunity contributed to cross-protection, CD4 and CD8 T cells were depleted from the heterosubtypic prime-boost immunized mice 2 days before and after challenge with Phil/H3N2 virus. Severe weight losses (>25%) and 0% survival rates were observed in both the CD4 and CD8 T cell-depleted groups. In contrast, the non-depleted vaccinated mice showed a moderate weight loss of about 11% and were 100% protected against Phil/H3N2 virus challenge (Figure 5C,D). These results suggested that protection against heterosubtypic Phil/H3N2 virus was dependent on both CD4 and CD8 T cells in the mice vaccinated with iSH/H7N9-iPR8/H1N1.

### 3.6. Heterosubtypic Prime-Boost Vaccination Controls Inflammatory Cytokines and Viral Replication after H3N2 Virus Challenge

**Figure 6:**
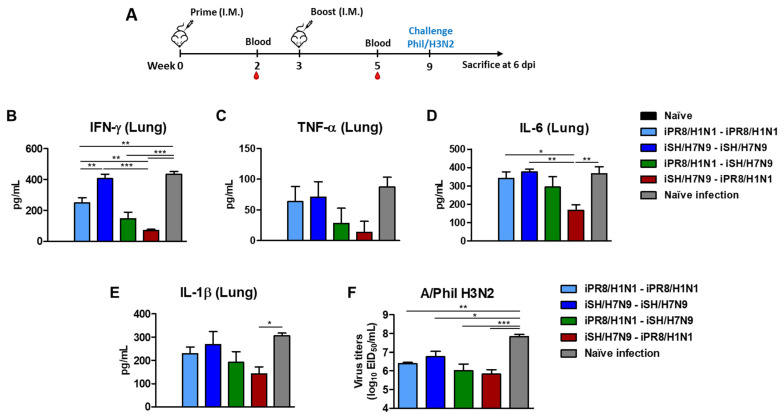
Heterosubtypic prime-boost vaccination controls inflammatory cytokines and lung viral loads. (**A**) Immunization scheme (*n* = 4 per group). Five to six weeks post-boost immunization, the mice were challenged with a lethal dose of Phil/H3N2 virus (6.7 × LD_50_, equivalent to 9.4 × 10^2^ EID_50_). (**B**) IFN- γ, (**C**) TNF-α, (**D**) IL-6, and (**E**) IL-1β cytokine levels in lung extracts at day 6 post-infection. (**F**) Lung viral titers as 50% egg infectious titers (EID_50_) at day 6 post-infection using embryonated chicken eggs. Statistical significance was calculated using one-way ANOVA and Tukey’s post-multiple comparison test. Error bars indicate the mean ± SEM. Lines under * symbols mark the comparing groups. *; *p* < 0.05, **; *p* < 0.01, ***; *p* < 0.001.

To further investigate the cross-protective efficacy, lung samples were collected on day 6 after Phil/H3N2 challenge to determine the levels of inflammatory cytokines (Figure 6A). The levels of inflammatory cytokines provided an additional barometer for assessing the protective efficacy of vaccination. The naïve infection control group showed the highest levels of IFN-γ, TNF-α, IL-6, and IL-1β cytokines in lung samples at 6 days after infection. In contrast, the induction of inflammatory cytokines (IFN-γ, IL-6) was more effectively prevented in the iSH/H7N9-iPR8/H1N1 group than in the homologous groups (Figure 6B–E). Additionally, lung viral titers were determined using embryonated chicken eggs (Figure 6F). The iPR8/H1N1-iSH/H7N9 and iSH/H7N9-iPR8/H1N1 groups showed significantly lower levels of virus titers (~100-fold lower) compared to the naïve infection group and ~10-fold lower viral titers than the homologous (iSH/H7N9-iSH/H7N9) vaccination group. These data suggested that heterosubtypic prime-boost vaccination would be effective in controlling viral loads and preventing severe inflammation.

### 3.7. Heterosubtypic Prime-Boost Vaccination Enhances IFN-γ-Producing Cells and HA Stalk-Specific Antibody-Secreting Cellular Responses after Phil/H3N2 Virus Challenge

**Figure 7:**
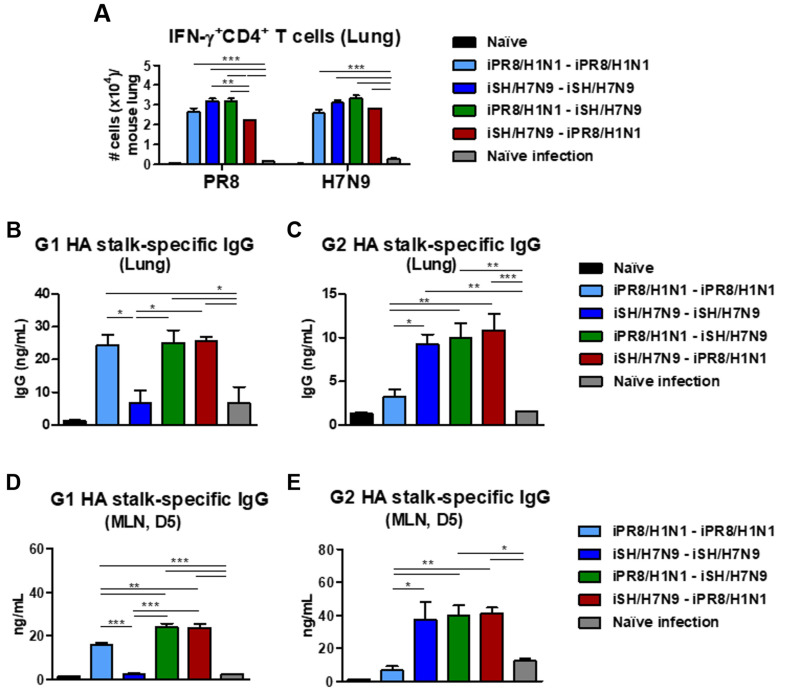
Cytokine-producing and stalk-specific IgG-secreting lung cellular responses after heterosubtypic prime-boost vaccination and Phil/H3N2 virus challenge. Immunization scheme (*n* = 4 per group) with the same vaccine groups as in Figure 6. (**A**) Flow cytometry data of IFN-γ-producing CD4^+^ T cells in lung samples at day 6 post-infection after stimulation with inactivated PR8/H1N1 or SH/H7N9 virus. (**B**) G1 and (**C**) G2 HA stalk-specific IgG levels in lung samples collected on day 6 post-challenge. (**D**) Groups 1 and (**E**) 2 HA stalk-specific IgG production from MLN cells harvested on day 6 post-infection, cultured in vitro for 5 days in the presence of G1 or G2 HA stalk proteins. Statistical significance was calculated using one-way ANOVA and Tukey’s post-multiple comparison test. Error bars indicate the mean ± SEM. *; *p* < 0.05, **; *p* < 0.01, ***; *p* < 0.001.

To determine the early cellular immune responses, lung cells harvested on day 6 after A/Phil/H3N2 virus infection were stimulated with iPR8/H1N1 or iSH/H7N9 virus. IFN-γ^+^ CD4^+^ T cell responses measured by intracellular cytokine staining upon stimulation with prime and boost inactivated virus strains revealed comparable levels of T cell responses in the homologous and heterologous prime-boost groups (Figure 7A). The iSH/H7N9-iPR8/H1N1 vaccination group showed a slightly lower level of lung IFN-γ^+^ CD4^+^ T cells upon PR8 stimulation than the other vaccine groups. All vaccination groups displayed significantly higher levels of IFN-γ^+^ CD4^+^ T cells (over 15-fold) than the naïve infection group (Figure 7A). These results suggested the induction of IFN-γ^+^ CD4^+^ T cells infiltrating into the lungs, which was induced by vaccination, as compared to naïve mice after infection.

The heterosubtypic prime-boost vaccination groups exhibited significantly higher levels of G1 and G2 HA stalk domain-specific IgG antibodies in lung extracts than the naïve infected mice (Figure 7B,C). As expected, the homologous prime-boost groups showed either G1 or G2 HA stalk-specific binding antibodies. Consistent with the patterns in lung extracts, after a 5-day culture of MLN cells, G1 HA stalk-specific IgG antibodies were induced at moderate levels in the heterosubtypic prime-boost groups but not in the iSH/H7N9-iSH/H7N9 group (Figure 7D,E). IgG antibodies to G2 HA stalk proteins were induced at moderate levels in the heterosubtypic prime-boost groups but not in the iPR8/H1N1-iPR8/H1N1 group. These data suggested that B cells can be effectively primed to generate G1 and G2 HA stalk domain-specific IgG responses in systemic MLNs and mucosal lung sites upon challenge after heterosubtypic prime-boost vaccination.

### 3.8. H1N1-H7N9 Heterosubtypic Prime-Boost Vaccination Confers Survival Protection against H5N1 and H9N2 Viruses

**Figure 8:**
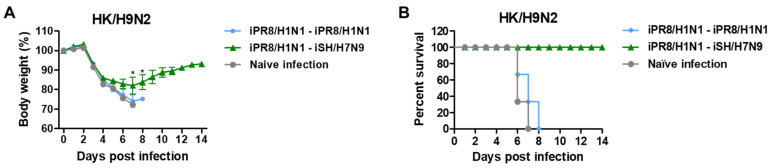
Heterosubtypic prime-boost vaccinations induce cross-protection against heterologous HK/H9N2 viruses. Immunization scheme (*n* = 3 per group) with the same vaccine groups as in Figure 6. (**A**) Body weight changes and (**B**) survival rates were monitored for 14 days after HK/H9N2 virus (6.7 × LD_50_, equivalent to 2.3 × 10^3^ EID_50_) challenge. Statistical significance was calculated using two-way ANOVA and Bonferroni’s post-multiple comparison test. Error bars indicate the mean ± SEM. *; *p* < 0.05 compared to the iPR8/H1N1-iPR8/H1N1 group.

To investigate whether heterosubtypic H1N1 and H7N9 prime-boost would induce broader cross-protection against different subtypes of viruses, mice were challenged with G1 HA HK/H9N2 virus at 6 weeks after boost immunization. The heterosubtypic iPR8/H1N1-iSH/H7N9 prime-boost group showed survival protection by displaying weight loss (~18%) and 100% survival rate against HK/H9N2 virus challenge as compared to the homologous iPR8/H1N1-iPR8/H1N1 and naïve infection groups that displayed weight losses of over 25% and 0% survival rates (Figure 8A,B).

To investigate whether adjuvants can enhance cross-protection, CpG + MPL (CpG/M) or QS-21 + MPL (QS/M) combination adjuvants were included in prime and boost vaccination in the heterologous iPR8/H1N1-iSH/H7N9 group. Both CpG/M and QS/M combination adjuvants slightly increased G1 and G2 HA stalk IgG antibodies as well as HAI titers against PR8/H1N1 and SH/H7N9 viruses without statistical significance, as compared to the groups without adjuvants (Supplementary Figure S2A–C). The inclusion of the CpG/M or QS/M adjuvant combination in the iPR8/H1N1-iSH/H7N9 vaccination induced less body weight losses (16% for CpG/M and 18% for QS/M) against rgH5N1 virus and 100% survival rates, compared to the unadjuvanted iPR8/H1N1-iSH/H7N9 (>22% weight loss), indicating that CpG/M or QS/M adjuvant in the heterosubtypic prime-boost vaccination slightly enhanced survival protection against rgH5N1 virus challenge (Supplementary Figure S3A,B). Taken together, these results further supported the effectiveness of heterologous prime-boost vaccination in conferring broader cross-protection with differential efficacy compared to repeat vaccination with the same vaccine.

### 3.9. Hetero-G1 H1N1 Prime-Boost Serum Is Effective in Providing Protection against G1 H5N1 Virus Compared to H7N9-H1N1 G2-G1 HA Virus Prime-Boost

**Figure 9:**
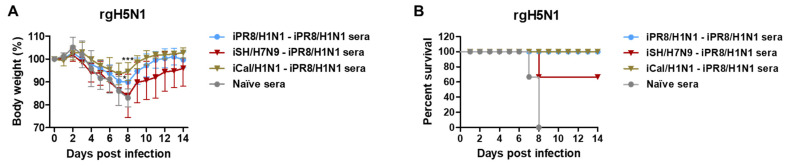
Immune sera from the prime-boost vaccination group inducing G1 HA stalk IgG antibodies contribute to protection against G1 HA rgH5N1 virus. (**A**) Body weight changes and (**B**) survival rates were monitored for 14 days in naïve mice (*n* = 3 per group) after inoculation with a mixture of rgH5N1 virus (3 × LD_50_, equivalent to 2.6 × 10^4^ EID_50_) and boost immune sera collected from the indicated groups. Statistical significance was calculated using two-way ANOVA and Bonferroni’s post-multiple comparison test. Error bars indicate the mean ± SEM. *; *p* < 0.05, ***; *p* < 0.001 compared to the naïve sera group in (**A**).

To further understand whether the protection conferred by immune sera was because of differences in hetero prime-boost immunizations with group-specific HA stalk antigens, naïve BALB/c mice were intranasally inoculated with a mixture of rgH5N1 virus at a lethal dose and antisera that were collected from vaccinated or naïve mice. Naïve sera did not provide protection against rgH5N1 virus, as evidenced by severe weight loss (> 25%) and 0% survival rate in naïve mice (Figure 9A,B). In contrast, immune sera from the hetero-G1 iCal/H1N1-iPR8/H1N1 group conferred protection in naïve mice with moderate weight loss (~6%) and 100% survival rate against G1 rgH5N1 virus. Notably, immune sera from the iPR8/H1N1-iPR8/H1N1 group conferred better protection (~10%) against G1 rgH5N1 virus than immune sera from the G2-G1 iSH/H7N9-iPR8/H1N1 group (>16% weight loss), although the difference was not statistically significant. These results suggested that immune sera from the groups that were vaccinated with G1 hetero viral antigens during prime and boost (iCal/H1N1-iPR8/H1N1) conferred better protection against G1 H5N1 virus than hetero-group HA prime-boost sera (iSH/H7N9-iPR8/H1N1).

## 4. Discussion

There is an important controversial issue regarding heterogeneity and reduced vaccine effectiveness in the current annual repeat influenza vaccination strategies [6,11,12,13,14,29,30,31]. In this study, we investigated whether heterologous prime-boost vaccination would induce more effective cross-protection than homologous repeat vaccination. Prime vaccination induced vaccine strain-specific HAI titers. A strategy of heterologous prime-boost vaccine combinations induced increased HAI activities against the prime virus strain as well as the heterologous boost virus and higher levels of group-specific HA stalk IgG antibody responses compared to those in prime sera. Heterologous prime-boost vaccination more effectively prevented severe weight loss after challenge with Phil/H3N2 virus than homologous repeat vaccination in the absence of serum HAI activity against the challenge virus. Boosting with vaccine strains different from the primed or pre-existing immune virus could be a more effective strategy of vaccination that might induce broader cross-protection. A prior clinical study in healthy adults reported that heterologous booster dose with H5N1 viral antigen (A/Turkey, clade 2.2.1) after priming with adjuvanted H5N1 antigen (A/Indonesia, clade 2.1.3.2) widened the cross-clade antibody responses to drift variants [32]. Also, heterologous prime-boost vaccination with European and North American H3N2 swine influenza virus (83% homology in the HA1 globular domain) induced neutralizing antibodies against both strains in pigs [33]. These prior studies are in support of a heterologous prime-boost vaccination strategy to enhance the breadth of cross-protection.

It is critical to better understand how prior immunity affects the quality of antibody responses to subsequent vaccination with the same or different influenza strains, particularly with respect to improving the efficacy of current influenza vaccination and preparing for pandemics. In contrast to vaccine strain-specific IgG antibody responses after the prime dose, boost vaccination induced broadly cross-reactive IgG antibodies binding to different virus strains, regardless of the strain used in boost vaccination. It was noted that A/Cal/H1N1 showed the least antigenicity against immune sera from different groups, which might be an advantage for this virus strain in avoiding host immunity and becoming a pandemic strain. The patterns of HAI titers were highly strain-specific and predictive, correlating with the different antigenic strains used for prime and boost vaccinations, compared to the binding IgG antibodies. HAI titers against A/PR8/H1N1 were significantly increased after heterologous prime-boost vaccinations when used either in prime or boost, comparable to prime vaccination with iPR8/H1N1. In addition, HAI titers against the different strains used as either prime or boost strains (iCal/H1N1-iPR8/H1N1, iHK/H3N2-iPR8/H1N1, iSH/H7N9-iPR8/H1N1) were similar or higher than those induced by prime vaccination with the same strain, regardless of the phylogenetic and antigenic distances (Supplementary Figure S1A–C). The results of heterologous prime-boost vaccination suggested weak evidence of the phenomenon of original antigenic sin (OAS), which induces antibody responses toward previously exposed influenza antigens while suppressing immune responses to closely related new antigens [34]. In contrast, this study demonstrated that prior priming with one strain did not interfere with inducing HAI titers against another strain while inducing higher HAI titers against a prior strain. It was also reported that original antigenic sin was not observed in humans and ferrets with prior influenza virus infection after exposure to the 2009 H1N1 pandemic virus [35]. The results in our study were found to be consistent with a clinical study that demonstrated the induction of higher levels of antibody responses by vaccines containing new strains compared to the vaccines containing the same strains used in the previous year [36]. Current recommendations for seasonal vaccination could be improved, especially when the vaccine antigenic composition is different from that of the previous winter season.

Pre-existing immune history shapes the profile of anti-influenza virus immune responses [34], but its impact on the protective efficacy of influenza vaccination is not well understood. Regardless of the vaccine strains used, heterologous prime-boost vaccinations were found to provide similar or more effective cross-protection than in the homologous prime-boost groups. For example, higher efficacies of cross-protection against Phil/H3N2 virus were observed in the heterologous prime-boost groups of iCal/H1N1-iPR8/H1N1, iHK/H3N2-iPR8/H1N1, iSH/H7N9-iPR8/H1N1, and iPR8/H1N1-iHK/H9N2. None of these heterologous groups showed cross-reactive HAI activity against Phil/H3N2 virus, suggesting non-HAI antibody immune-mediated protection. HA stalk-specific IgG antibodies were reported to be independently correlated with protection against different subtype viruses [37]. Group 2 (G2) HA stalk-specific IgG levels were substantially induced by these heterologous vaccinations except for iCal/H1N1-iPR8/H1N1, which induced group 1 (G1) HA-specific stalk antibodies at high levels. It is notable that heterologous iHK/H9N2-iPR8/H1N1 vaccination induced both G1 and G2 stalk-specific IgG antibodies even though both H1 and H9 are phylogenetically categorized into G1 HA. Preparation of stabilized recombinant stalk protein vaccines has been challenging, and the efficacy of G2 stalk vaccines has been low, particularly with heterologous viruses [38,39,40]. An alternative strategy to develop a universal influenza vaccine inducing HA stalk-specific antibodies was demonstrated by using reassortant viruses with chimeric HA of different subtype head but the same HA stalk domain [41,42,43]. This study suggested a moderate correlation between levels of HA stalk antibodies and cross-protection (Figure 4D), and antisera of iSH/H7N9-iPR8/H1N1 vaccination conferred protection against A/Phil/H3N2 virus in naïve mice.

The levels of stalk-specific IgG antibodies alone would not explain the efficacy of cross-protection by certain heterologous vaccinations. The iCal/H1N1-iPR8/H1N1 group could induce cross-protection against Phil/H3N2, preventing severe weight loss despite inducing low levels of G2 stalk antibodies. The depletion of CD4 or CD8 T cells in mice vaccinated with iSH/H7N9-iPR8/H1N1 resulted in the abrogation of cross-protection, suggesting an important role of T cell immunity. Heterologous prime-boost vaccination-induced IgG antibody responses binding to challenge whole virus would not correlate with predictive efficacy between heterologous and homologous vaccination regime as shown by IgG responses and protection against HK/H9N2 virus and rgH5N1 virus (Table 2, Figure 8 and Figure 9). It was unexpected that iCal/H1N1 homo prime boosting resulted in more severe weight loss after A/Phil/H3N2 virus challenge than the naïve mouse infection control. We do not understand the underlying pathological mechanisms of influenza disease in mice with certain homo prime-boost vaccination after heterosubtypic challenge. It is speculated that limited breadth of pre-existing immunity might facilitate accumulation of immune complexes and immune pathology but might be ineffective in clearing lung viral loads after exposure to an antigenically distant pathogenic virus. T cell responses, upon stimulation with vaccine virus strains at day 6 post-challenge, did not significantly differ between heterologous and homologous vaccination regimes (Figure 7A), partially due to differential lung viral loads. It might be more insightful to study the challenge virus-specific T cell responses before and after challenge in future studies.

Clinical studies on repeat annual influenza vaccination indicate some concerns such as diminished B-cell responses, reduction in antibody affinity maturation, and lower vaccine efficacy against mismatch strains [12,13,14,15,36]. In summary, heterologous vaccinations could induce enhanced and broader antibody responses such as HAI titers against different prime and boost strains as well as G1 and G2 HA stalk-specific IgG responses. Improved cross-protection was observed with various heterologous prime-boost vaccinations compared to homologous prime-boost vaccinations. Both humoral and cellular immunities were independently important and might have contributed to cross-protection by heterologous prime-boost vaccinations. Vaccination with antigenically different vaccine strains rather than repeat vaccination with the same antigen might provide a strategy for improving the breadth of protection. There were limitations in this study, such as not fully recapitulating the complex history of pre-existing immunity and the current quadrivalent influenza vaccine for humans. Another weakness of this study was the lack of data determining cellular immune responses specific to the challenge virus before and after challenge. Comparing vaccination with multi-valent vaccine components versus sequential heterologous influenza vaccination remains to be determined, which will be important for future studies.

## Figures and Tables

**Table 1:** Virus- and HA stalk-specific IgG antibody responses in immune sera collected two weeks after prime vaccination with different inactivated viruses. The prime vaccine groups are the same as in the Figure 1A legend. The relative comparison of IgG antibody responses is presented as an average of IgG concentrations in prime sera.

Vaccine Groups	Virus-Specific IgG (µg/mL)						HA Stalk-Specific IgG (µg/mL)	
Prime	iPR8/H1N1	iCal/H1N1	iHK/H3N2	iIndo/H5N1	iSH/H7N9	iHK/H9N2	G1 Stalk	G2 Stalk
**iPR8/H1N1**	10.56	1.24	8.38	1.43	5.20	2.82	1.81	0.10
**iCal/H1N1**	1.43	7.98	2.43	0.84	1.43	1.04	1.06	0.10
**iHK/H3N2**	6.00	1.24	28.82	2.03	8.38	3.82	0.14	0.48
**iIndo/H5N1**	6.00	1.04	9.97	15.13	7.98	4.21	0.36	0.12
**iSH/H7N9**	4.41	1.04	8.78	1.83	10.36	3.02	0.14	0.64
**iHK/H9N2**	3.02	1.43	5.40	0.84	1.83	8.38	0.36	0.18
**Naïve**	0.08	0.08	0.10	0.08	0.08	0.08	0.14	0.18

**Table 2:** Comprehensive summary of different virus- and HA stalk-specific IgG antibody responses in immune sera collected two weeks after homologous and heterologous prime-boost vaccinations. The boost vaccination groups are the same as described in the Figure 1B legend and vaccination scheme. The comparison of IgG antibody responses is presented as an average of IgG concentrations in boost sera.

Vaccine Groups		Virus-Specific IgG (µg/mL)						HA Stalk-Specific IgG (µg/mL)	
Prime	Boost	iPR8/ H1N1	iCal/ H1N1	iHK/ H3N2	iIndo/ H5N1	iSH/ H7N9	iHK/ H9N2	G1 Stalk	G2 Stalk
**iPR8/H1N1**	**iPR8/H1N1**	155.2	28.2	149.3	113.6	91.7	79.8	14.1	4.0
**iPR8/H1N1**	**iCal/H1N1**	79.8	34.2	103.6	85.8	67.9	50.1	20.9	3.6
**iCal/H1N1**	**iPR8/H1N1**	40.1	44.1	42.1	40.1	26.3	22.3	22.9	3.8
**iPR8/H1N1**	**iHK/H3N2**	105.6	30.2	250.5	161.2	121.5	73.9	3.8	10.4
**iHK/H3N2**	**iPR8/H1N1**	83.8	24.3	171.1	99.7	73.9	48.1	13.1	12.1
**iPR8/H1N1**	**iIndo/H5N1**	67.9	18.3	97.7	125.5	73.9	44.1	25.8	4.6
**iIndo/H5N1**	**iPR8/H1N1**	89.7	14.3	105.6	115.5	73.9	28.2	18.7	2.0
**iPR8/H1N1**	**iSH/H7N9**	99.7	30.2	210.8	177.0	131.4	44.1	8.4	9.2
**iSH/H7N9**	**iPR8/H1N1**	139.3	22.3	177.0	139.3	127.4	38.2	18.5	5.4
**iPR8/H1N1**	**iHK/H9N2**	77.8	24.3	196.9	121.5	87.8	58.0	3.8	4.0
**iHK/H9N2**	**iPR8/H1N1**	123.5	16.3	155.2	137.4	111.6	52.0	22.1	7.2
**Naïve**		0.05	0.10	0.08	0.10	0.10	0.08	0.08	0.14

**Table 3:** Summary of HAI titers against homologous, heterologous, and heterosubtypic viruses in immune sera collected two weeks after prime vaccination. HAI titers in prime sera were determined as represented in Figure 2A data.

Vaccine Groups	HAI Titers against Different Virus Strains						
Prime	PR8/H1N1	Cal/H1N1	HK/H3N2	Indo/H5N1	SH/H7N9	HK/H9N2	Phil/H3N2
**iPR8/H1N1**	1:64	-	-	-	-	-	-
**iCal/H1N1**	-	1:64	-	-	-	-	-
**iHK/H3N2**	-	-	1:256	-	-	-	-
**iIndo/H5N1**	-	-	-	1:32	-	-	-
**iSH/H7N9**	-	-	-	-	1:64	-	-
**iHK/H9N2**	-	-	-	-	-	1:256	-
**Naïve**	-	-	-	-	-	-	-

**Table 4:** Summary of HAI titers in immune sera collected two weeks after homologous and heterologous prime-boost vaccinations. HAI titers in boost sera were determined as represented in Figure 2B data.

Vaccine Groups		HAI Titers against Different Virus Strains						
Prime	Boost	PR8/ H1N1	Cal/ H1N1	HK/ H3N2	Indo/ H5N1	SH/ H7N9	HK/ H9N2	Phil/ H3N2
**iPR8/H1N1**	**iPR8/H1N1**	1:512	-	-	-	-	-	-
**iPR8/H1N1**	**iCal/H1N1**	1:256	1.64	-	-	-	-	-
**iCal/H1N1**	**iPR8/H1N1**	1:256	1:256	-	-	-	-	-
**iPR8/H1N1**	**iHK/H3N2**	1:256	-	1:1024	-	-	-	-
**iHK/H3N2**	**iPR8/H1N1**	1:256	-	1:512	-	-	-	-
**iPR8/H1N1**	**iIndo/H5N1**	1:256	-	-	1:64	-	-	-
**iIndo/H5N1**	**iPR8/H1N1**	1:512	-	-	1:64	-	-	-
**iPR8/H1N1**	**iSH/H7N9**	1:256	-	-	-	1:256	-	-
**iSH/H7N9**	**iPR8/H1N1**	1:512	-	-	-	1:512	-	-
**iPR8/H1N1**	**iHK/H9N2**	1:256	-	-	-	-	1:256	-
**iHK/H9N2**	**iPR8/H1N1**	1:512	-	-	-	-	1:512	-
**Naïve**		-	-	-	-	-	-	-

## Data Availability

All data are available in the main text or Supplementary Materials.

## Supplementary Materials

The following supporting information can be downloaded at: https://www.mdpi.com/article/10.3390/vaccines11071209/s1, Figure S1: Phylogenetic tree and amino acid (aa) homology between the HA of A/PR8/H1N1 and HA of other influenza virus strains, Figure S2: Adjuvanted heterosubtypic prime-boost vaccinations increase HA stalk-specific IgG antibodies, Figure S3: Adjuvanted heterosubtypic prime-boost vaccinations improve survival protection against rgH5N1 virus.
