# Supplementary material for: Heterologous Prime-Boost Vaccination with Inactivated Influenza Viruses Induces More Effective Cross-Protection than Homologous Repeat Vaccination
Source: Vaccines (Basel). 2023 Jul 6;11(7):1209. doi: 10.3390/vaccines11071209 (PMC10386405; doi:10.3390/vaccines11071209)
Supplement: Supplementary file 1 [file vaccines-11-01209-s001.zip › vaccines-2402083-supplementary.pdf]

Supplementary Information for

Heterologous prime-boost vaccination with inactivated influenza viruses induces more effective cross-protection than homologous repeat vaccination

Supplementary Figure S1

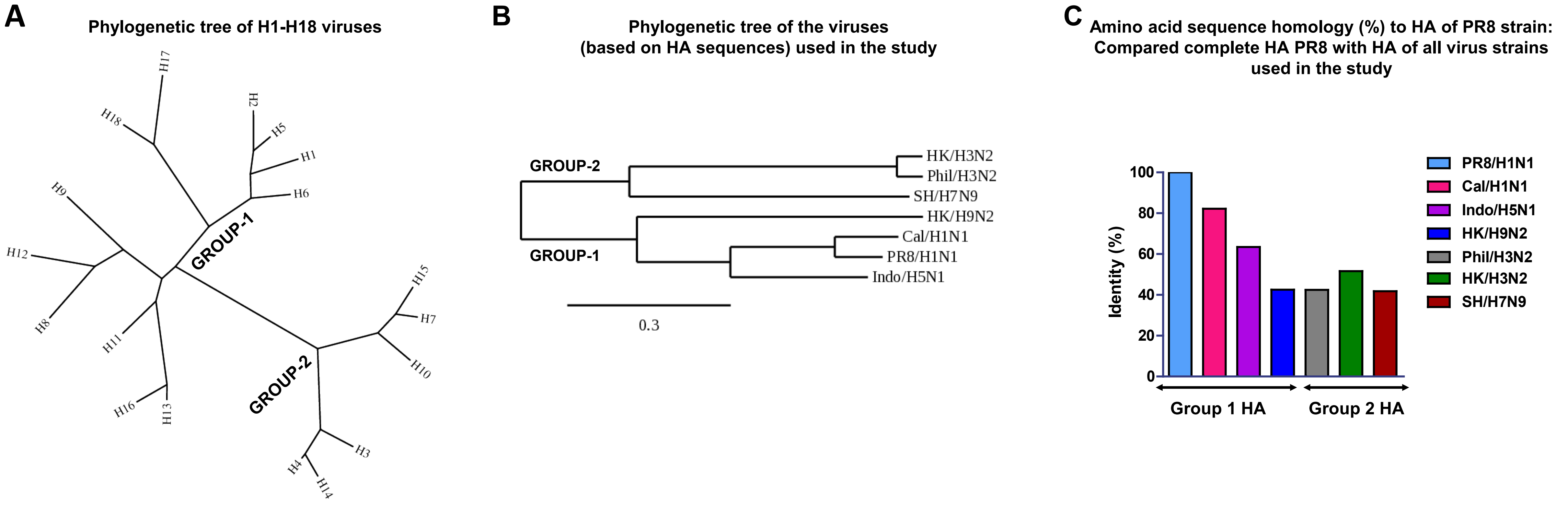

Supplementary Figure S1. Phylogenetic tree and amino acid (aa) homology between the HA of A/PR8/H1N1 and HA of other influenza virus strains. (A) A phylogenetic tree was constructed based on the aa sequences of the HA genes in H1-H18 influenza viruses obtained from GenBank. (B) Phylogenetic tree based on the aa sequences (obtained from GenBank) of the HA genes of the influenza viruses used in the study. Influenza A (H1-H18) HA sequences were obtained from GenBank: NC_002017 H1 HA (YP_163736) for A/Puerto Rico/8/1934 (PR8/H1N1), NC_026433A H1 HA (YP_009118626) for A/California/07/2009 (Cal/H1N1), L11125 for H2 HA of A/Berkeley/1968 (AAA43089), CY080523 H3 HA (ADV76673) for A/Hong Kong/1-10-MA21-1/1968 (HK/H3N2), IVU08858 H3 HA (AAA18781) for A/Philippines/2/82 (Phil/H3N2), MT421019 for H4 HA (QJI55045), CY116646 for H5 HA of A/Indonesia/5/2005 (Indo/H5N1) (AFM78567), CY166897 for H6 HA (AHL82551), KC853228 for H7 HA of A/Shanghai/2013 (SH/H7N9) (AGI60292), CY097534 for H8 HA (AEM75966), KF188366 for H9 HA of A/chicken/Hong Kong/G9/1997 (HK/H9N2) (AGO17823), MF613851 for H10 HA (ASV60666), CY191275 for H11 HA (AKF35393), CY133357 for H12 HA (AGE03167), CY054300 for H13 HA (ADB46159), MK327694 for H14 HA (AZQ09016), KP087869 for H15 HA (AIY68624), CY177441 for H16 HA (AHM98288), CY103892 for H17 HA (AFC35438), CY125945 for H18 HA (AGX84934). The complete HA and HA2 sequences were used to construct the phylogenetic trees using the Clone Manager program and online tools (http://www.phylogeny.fr). (C) The amino acid (aa) sequence homology between the HA of A/PR8/H1N1 and HA of other influenza virus strains. The HA sequences of the viruses used in this study were obtained from GenBank to analyze the aa sequence identity. The percentages of the aa homology of the HA sequence of A/PR8/H1N1 virus with the HA sequences of other virus strains were calculated using Needle (EMBOSS; EMBL-EBI).

Supplementary Figure S2

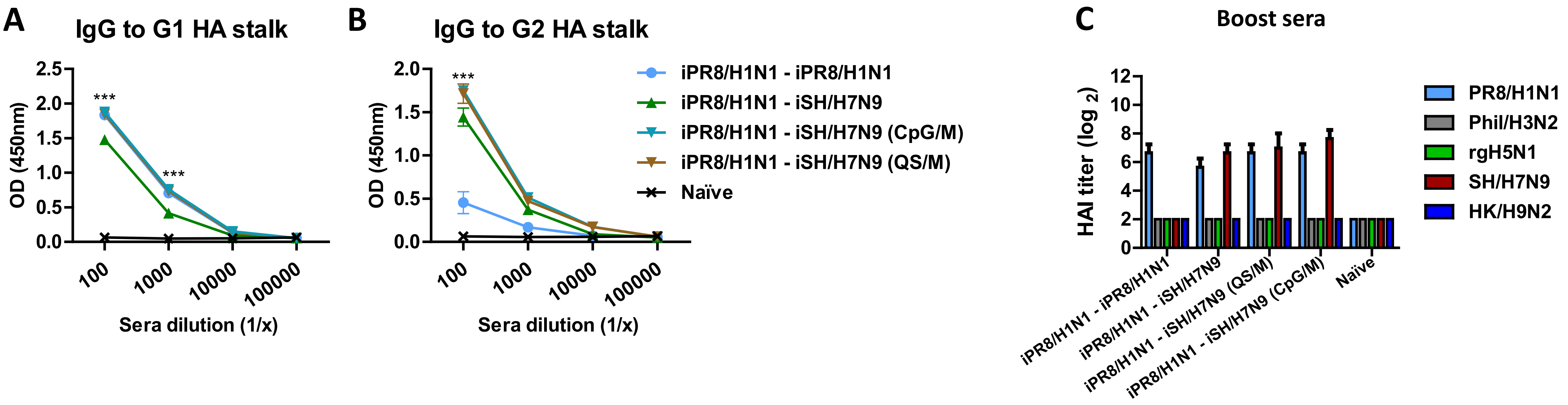

Supplementary Figure S2. Adjuvanted heterosubtypic prime-boost vaccinations increase HA stalk-specific IgG antibodies. Pure Quillaja saponin, QS-21, and monophosphoryl lipid A (MPL) adjuvants were purchased from Desert King International (San Diego, CA, USA) and Sigma Aldrich (St. Louis, MO), respectively, and dissolved in dimethyl sulfoxide (DMSO) following the manufacturer's protocol. Mouse-specific oligodeoxynucleotide (ODN) with CpG motifs (ODN1826, 5′-TCC ATG ACG TTC CTG ACG TT-3′) was synthesized by Integrated DNA Technologies (IDT, Coralville, IA). The lyophilized CpG was resuspended in ultra-pure water. Immunization scheme (n = 3 per group). BALB/c mice were intramuscularly primed with 5 μg of a specific strain of inactivated influenza virus and then boosted with 5 μg of a homologous or heterosubtypic inactivated virus (H1N1, or H7N9) with or without adjuvants CpG (4 μg)/ MPL (1 μg)/mouse (CpG/M) or QS-21 (10 μg)/ MPL (1 μg)/mouse (QS/M). (A) G1 and (B) G2 HA stalk-specific IgG antibody levels in boost sera. (C) HAI titers against different viruses as indicated in boost sera. Statistical significance was calculated using two-way ANOVA and Bonferroni's post-multiple comparison test. Error bars indicate the mean ± SEM. ***; $p < 0.001$ compared to the iPR8/H1N1 – iSH/H7N9 group.

Supplementary Figure S3

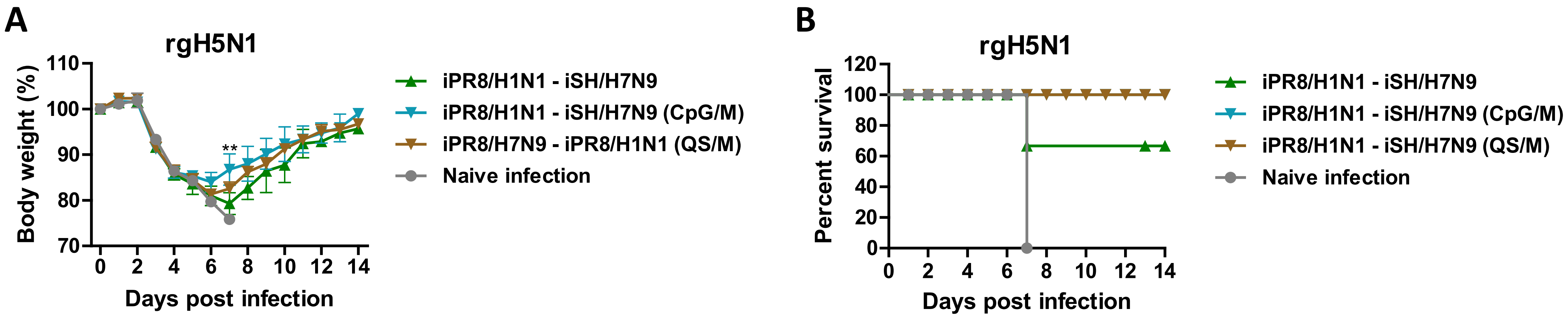

Supplementary Figure S3. Adjuvanted heterosubtypic prime-boost vaccinations improve survival protection against rgH5N1 virus. (A) Body weight changes and (B) survival rates were monitored for 14 days after rgH5N1 virus ($5 \times LD_{50}$, equivalent to 7.9×10^4 EID_{50}) challenge. Statistical significance was calculated using two-way ANOVA and Bonferroni's post-multiple comparison test. Error bars indicate the mean ± SEM. **; $p < 0.01$, compared to the iPR8/H1N1 – iSH/H7N9 group.
